# Segmentally Variable Genes:A New Perspective on Adaptation

**DOI:** 10.1371/journal.pbio.0020081

**Published:** 2004-04-13

**Authors:** Yu Zheng, Richard J Roberts, Simon Kasif

**Affiliations:** **1**Bioinformatics Graduate Program, Boston UniversityBoston, MassachusettsUnited States of America; **2**New England Biolabs, BeverlyMassachusettsUnited States of America; **3**Department of Biomedical Engineering, Boston UniversityBoston, MassachusettsUnited States of America

## Abstract

Genomic sequence variation is the hallmark of life and is key to understanding diversity and adaptation among the numerous microorganisms on earth. Analysis of the sequenced microbial genomes suggests that genes are evolving at many different rates. We have attempted to derive a new classification of genes into three broad categories: lineage-specific genes that evolve rapidly and appear unique to individual species or strains; highly conserved genes that frequently perform housekeeping functions; and partially variable genes that contain highly variable regions, at least 70 amino acids long, interspersed among well-conserved regions. The latter we term segmentally variable genes (SVGs), and we suggest that they are especially interesting targets for biochemical studies. Among these genes are ones necessary to deal with the environment, including genes involved in host–pathogen interactions, defense mechanisms, and intracellular responses to internal and environmental changes. For the most part, the detailed function of these variable regions remains unknown. We propose that they are likely to perform important binding functions responsible for protein–protein, protein–nucleic acid, or protein–small molecule interactions. Discerning their function and identifying their binding partners may offer biologists new insights into the basic mechanisms of adaptation, context-dependent evolution, and the interaction between microbes and their environment.

## Abstract 2

Segmentally variable genes show a mosaic pattern of one or more rapidly evolving, variable regions. Discerning their function may provide new insights into the forces that shape genome diversity and adaptation

## Introduction

Microbes occupy almost every habitable niche in the biosphere, highlighting their enormous capability for adaptation and survival. This adaptive ability has been refined during millennia of evolution and has resulted in genes that evolve at very different rates. Some, such as housekeeping genes that code for the essential biochemical functions of the organism, are now evolving rather slowly. Others that have to defend against potentially lethal attack by viruses or toxins and adapt to varying environmental conditions, often evolve more rapidly (Murphy 1993; Moxon and Thaler 1997; Jordan et al. 2002). Pathogenic microbes, for example, face stringent tests of their adaptive potential because of the escalating efficiency of the host-defense mechanisms (Moxon and Thaler 1997). In the arms race between pathogens and their hosts, both sides try to improve their overall fitness by deploying sophisticated strategies to generate genetic variability (Elena and Lenski 2003). Sequence divergence during rapid evolution can take many forms. Some genes change throughout their entire sequences, resulting in apparently lineage-specific genes that lack clear similar sequences in current versions of GenBank. Others show a mosaic pattern of one or more variable regions interspersed within conserved regions. This latter group is the subject of this paper and we refer to them as segmentally variable genes (SVGs). For the purpose of the current analysis, we define such variable regions as having a minimum length of 70 amino acids, which would permit them to fold into independent domains. This distinguishes them from most nonfunctional interdomain segments, which are usually shorter and whose principal function depends on length rather than specific sequence content.

**Figure 1:**
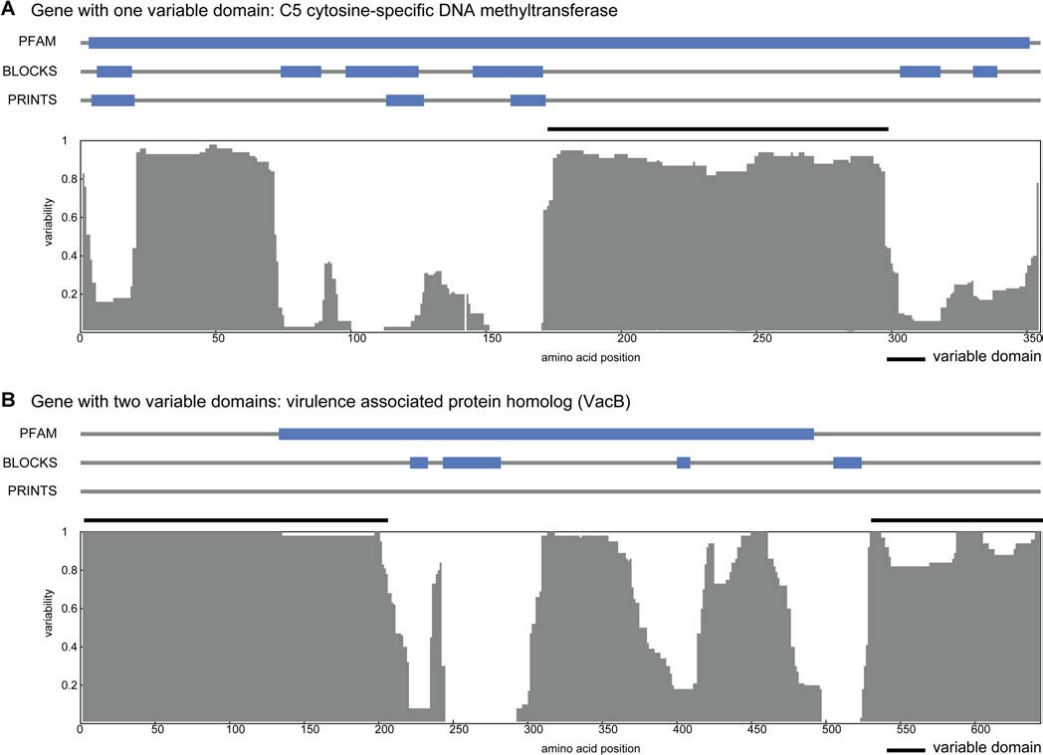
Variability Profile for Typical SVGs Blocks in the lines are conserved subsequences identified using the Pfam, BLOCKS, and PRINTS databases. In the variability profile, the x-axis is the amino acid position and the y-axis is the variability index (see Materials and Methods). Variable domains are marked by the black lines over the graph. (A) Cytosine-specific DNA methyltransferase of 355 amino acid long in H. pylori. Notice the variable domain in the middle and the variable segment in its N-terminal region, which is shorter than 70 amino acids and has no known function. (B) Virulence-associated protein homolog (VacB) of 644 amino acid long in H. pylori. It has two variable domains at the N- and C-termini.

An example of an SVG family is provided by the cytosine-5 DNA methyltransferases (Posfai et al. 1989). These enzymes typically form parts of restriction-modification systems, which are key components of an important bacterial defense mechanism to protect against phage attack and other unwanted infiltration of foreign DNA (Cheng 1995). These methyltransferases catalyze the addition of a methyl group from S-adenosylmethionine to the 5-position of cytosine and contain a highly variable region of more than 90 amino acids that is responsible for specific DNA sequence recognition (Figure 1A; Posfai et al. 1989; Cheng 1995; Lange et al. 1996). A detailed examination of the three-dimensional (3D) structure of the variable region suggests that it folds into an independent domain, which has been shown to bind to DNA (Cheng et al. 1993). The flanking sequences are highly conserved because they are responsible for the chemistry of methylation, which is common to all members of the family. Variability in this family has arisen because there is a need for great variation in the DNA sequences being recognized so that the specific pattern of methylation becomes a marker to distinguish innate DNA from foreign DNA.

To the best of our knowledge, there has been no systematic attempt to identify, catalog, and classify similar SVGs in the sequenced microbial genomes. Nor has any attempt been made to find potentially common functions among genes displaying this property. Since it is known that many genes involved in defense mechanisms, such as the DNA methyltransferases and the antigens exposed on the surface of bacteria, show such variability (Roche et al. 2001), it is tempting to speculate that one might identify host-defense genes based on this property. Thus, the regional variability might reflect the influence of diversifying selection pressure that could come from constant interaction with other fast-evolving molecules in the environment. Could such genes be the predominant members of the SVG families? Or do other genes, such as those involved in basic energy metabolism and synthesis, show similar variability? In this paper we provide an initial systematic analysis. We describe our findings about the distribution of SVGs and the potential function achieved by segmental variability.

## Results

### Classification of Genes into Three Broad Groups

We carried out a classification of the genes in 43 fully sequenced microbial genomes (see Table S1 for a full name list). A Web site (http://geneva.bu.edu) is also provided with results for several selected genomes, including *Escherichia coli, Helicobacter pylori, Neisseria meningitidis,* and several others. Each gene is accompanied with schematic diagrams from Pfam (Bateman et al. 2002), BLOCKS (Henikoff et al. 1999), PRINTS (Attwood et al. 2003), and the nongapped BLAST (Altschul et al. 1990) analyses.

For each genome, the full proteome is compared with the nonredundant GenBank sequence set using nongapped BLAST (see Materials and Methods for the parameters used). Based on the degree of conservation or divergence among similar genes in different species, we classify them into three broad groups. Lineage-specific genes are defined as genes with no significantly similar hits from other species in the current GenBank (*E*-value cutoff, 1*E*-5). SVGs are defined as genes containing at least one highly variable region, containing more than 70 amino acids, interspersed among well-conserved regions. In any single SVG family, the length of the variable region can differ only within a certain range (see Materials and Methods for more details). In this paper, regions are considered to be variable if no sequence similarity can be detected against possible homologous genes, where the overall homology is determined by the conserved portions. The rest of the genes in the genome are considered as fully conserved genes. Naturally, this initial soft classification is somewhat dependent on specific thresholds and will be biased by the current state of GenBank and the quality of the annotation.

**Figure 2:**
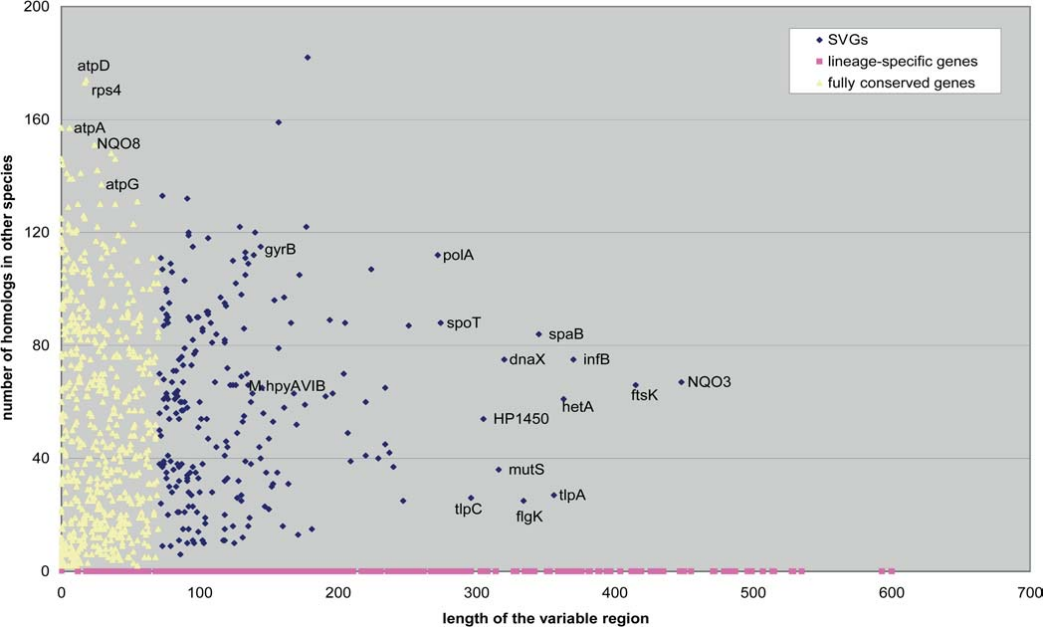
Classification of Three Groups of Genes from a Single Genome, H. pylori, in 2D Space The x-axis is the length of the variable region and the y-axis is the number of possible homologs a gene has from a BLAST search. The variable region length for a lineage-specific gene is defined as the length of the gene so that they naturally cluster onto the x-axis. Multiple variable regions in one gene are represented separately.

In Figure 2 we show a scatter plot of the three classes of genes in the H. pylori genome in two-dimensional (2D) space, where the x-axis shows the length of the variable region and the y-axis shows the number of possible homologs of each gene. Lineage-specific genes (filled square in Figure 2) by definition naturally cluster on the x-axis. Most of the genes in this group are still annotated as unknown. A few genes with annotated functions in this group, such as the outer-membrane protein family in H. pylori (Tomb et al. 1997), only appear in this organism and contribute to its unique biology. A second group contains fully conserved genes (filled triangle in Figure 2) with only short variable regions. It is in this class that most “housekeeping” genes fall. Examples include the subunits of ATP synthetase F1 (atpD, atpA, atpG) and ribosomal proteins such as rps4 (Figure 2), etc. The third group contains the SVGs (filled diamond in Figure 2). A few examples in this group are labeled with their names in Figure 2 and will be discussed later. In Table 1 we list the number of genes in each category for a representative set of microbial genomes (see Table S1 for a full list).

**Table 1:**
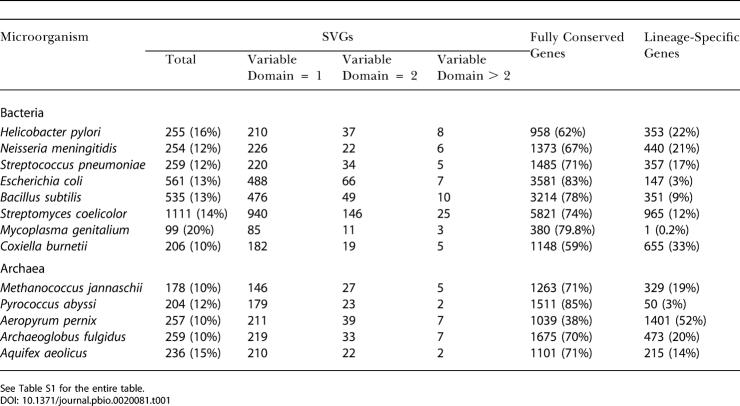
Classification of Genes into Three Broad Categories for a Representative Set of Microbial Genomes

See Table S1 for the entire table

SVGs are subdivided into different types depending on whether they have one, two, or more variable regions. The number of genes with a single variable region is much larger than the number of genes with multiple ones. In Figure 1A we show the variation profile of an SVG containing one variable region. The variation profile is displayed together with conserved subsequences identified using the Pfam (Bateman et al. 2002), BLOCKS (Henikoff et al. 1999), and PRINTS (Attwood et al. 2003) databases. This gene is the cytosine-specific DNA methyltransferase, M.HpyAVIB, from H. pylori. The variability lies in its DNA recognition domain (approximately 140 amino acids), which in this case recognizes the DNA sequence CCTC (Lin et al. 2001). In Figure 1B we give an example with two variable regions. It is the virulence-associated protein homolog VacB from H. pylori, which has variable regions at both its N-terminus (approximately 200 amino acids) and C-terminus (approximately 100 amino acids). *VacB* has been shown to encode a 3′–5′ exoribonuclease and is necessary for expression of virulence (Cheng and Deutscher 2002). The conserved central region (approximately 400 amino acids (Pfam domain: RNB) defines a group of homologs distributed in a number of microbial genomes (Zuo and Deutscher 2001). Note that the C-terminal region is variable, and its E. coli homolog contains RNA-binding motifs (Zuo and Deutscher 2001). Although the detailed physiological roles of VacB remain unknown (Cheng and Deutscher 2002), the variable regions may contribute to the determination of substrate specificity of VacB in the RNA quality-control process that eliminates defective ribosomal RNA (rRNA) molecules in different species.

The number of SVGs increases as genome sizes vary, from 0.5 MB*(Mycoplasma genitalium*) to 8.6 MB*(Streptomyces coelicolor*) (Table 1). For most microorganisms included, the proportion of SVGs varies in the range of 10%–20%. The number of lineage-specific genes, on the other hand, does not appear to correlate with the genome size. Instead, it is influenced by the content of the database. For instance, a “minimal” genome, M. genitalium, has a relatively high content of SVGs (20%) and a low percentage of lineage-specific genes (0.2%). However, when a closely related species, M. pneumoniae, is excluded from the database, its proportion of lineage-specific genes rises to 14%, while the proportion of SVGs remains unchanged. In general, the genomic proportion of SVGs is less affected by the database content.

### Case Studies of SVGs and Functional Implication of Variability

In the following sections, we have selected several SVG families to demonstrate the functional implication of segmental variability.

#### Outer-membrane signal transduction genes/sensor histidine kinases

**Figure 3:**
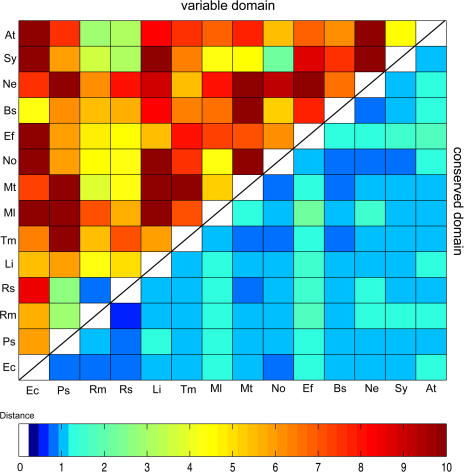
2D Representation of the Distance Matrix Computed from the Variable and Conserved Domains in a Group of Similar HKs The upper triangle shows the variable domains, the lower one the conserved domains. Amino acid sequence distances are calculated by the PROTDIST program using the Dayhoff PAM matrix. The sequence from each species is the best match (*E*-value < 1*E*-10) in that genome to the query E. coli gene. Abbreviations for organisms: Ec, Escherichia coli K12; Ps, Pseudomonas syringae pv. syringae B728a; Rm, Ralstonia metallidurans; Rs, Ralstonia solanacearum; Li, Listeria innocua; Tm, Thermotoga maritime; Ml, Mycobacterium leprae; Mt, Mycobacterium tuberculosis CDC1551; No, *Nostoc* sp. PCC 7120; Ef, Enterococcus faecalis; Bs, Bacillus subtilis; Ne, Nitrosomonas europaea; Sy, *Synechococcus* sp. PCC 7942; At, Agrobacterium tumefaciens. The PROTDIST program is included in the PHYLIP software package version 3.5 (Felsenstein 1989).

In prokaryotes, two-component signal-transducing systems are common and consist of a histidine kinase (HK) and a response regulator. Most HKs are membrane-bound, homodimeric proteins with an N-terminal periplasmic sensing domain and a C-terminal cytoplasmic kinase domain. HKs usually possess a highly variable sensing domain (usually over 150 amino acids), while the cytoplasmic kinase domain is quite conserved. By diversifying the sensing domain, microorganisms can develop different two-component modules to respond to different signals and interact with small molecules from the exterior. Figure 3 displays the distance matrix calculated from the sensing domains and the kinase domains from a group of highly similar HK genes. As shown in Figure 3, sensing domains are much more diverse than the kinase domains. Moreover, the two regions show distinct clustering patterns, of which only the one for the conserved kinase domains is close to the phylogenetic relationship inferred from 16S rRNA sequences (data not shown). Significant homologies in the sensing regions can only be found in closely related species (e.g., Ralstonia solanacearum [Rs] and Ralstonia metallidurans [Rm] in Figure 3), suggesting rapid divergence after speciation. Other sensor genes involved in cell motility*,* e.g., genes encoding methyl-accepting chemotactic protein (MCP) (see *tlpA, tlpC* in Figure 2), are also highly variable in their N-terminal domains. In several bacteria*,* e.g., Vibrio cholerae, there is a greater number of segmentally variable MCP genes (approximately 40) than in other genomes (see the gene list of V. cholerae at http://geneva.bu.edu), which must correspond to its expanded ability to detect different chemical signals and find favorable environments. Although a few conserved motifs have been detected in the sensing region (Galperin et al. 2001), the exact sensing signals for most prokaryotic HKs are unknown.

#### Transporter genes and outer-membrane proteins

The biggest family of SVGs is cell envelope-related, including the ATP-binding cassette transporters (ABC transporters), outer-membrane proteins, and virulence-related gene products. For membrane proteins, since part of their sequences are exposed to the outside of the cell and interact directly with the environment, one might hypothesize that the variable portions have evolved rapidly to deal with the changing environmental conditions.

ABC transporters are essential for microorganisms because they import nutrients into the cell and export noxious substances and toxins out of the cell. A typical ABC transporter gene in a prokaryote genome has a conserved ATPase domain (approximately 150 amino acids) and a large (over 300 amino acids) variable integral membrane domain. Two examples from this group are the multidrug-resistance genes *hetA* and *spaB* shown in Figure 2. It is known that substrates interact with the specific binding sites inside the membrane domain (Holland and Blight 1999), which suggests that the variability in the membrane domain may have to do with substrate selectivity or with different transport kinetics. Moreover, outer-membrane transporters are binding targets for bacteriophages and bacterial toxins. For example, the vitamin B12 transporter BtuB (614 amino acids) is the receptor for bacteriophage BF23 and E-colicin (Bradbeer et al. 1976; Mohanty et al. 2003). The crystal structure of BtuB in E. coli has been solved (Chimento et al. 2003). The variable region in E. coli BtuB overlaps with the 22-strand β-barrel (position 150–360), while the N-terminal hatch domain (position 6–132) and the extreme C-terminal TonB-box domain (position 550–614) are conserved among many homologs (Figure S1). The extracellular loops between contiguous strands in the β-barrel are displayed outside the cell (Chimento et al. 2003) and possibly serve as receptor sites for bacteriophages and toxins. The variability in these loops may be driven by attempts to defend against bacteriophages and interaction with different bacterial toxins.

#### DNA/RNA-processing enzymes

**Figure 4:**
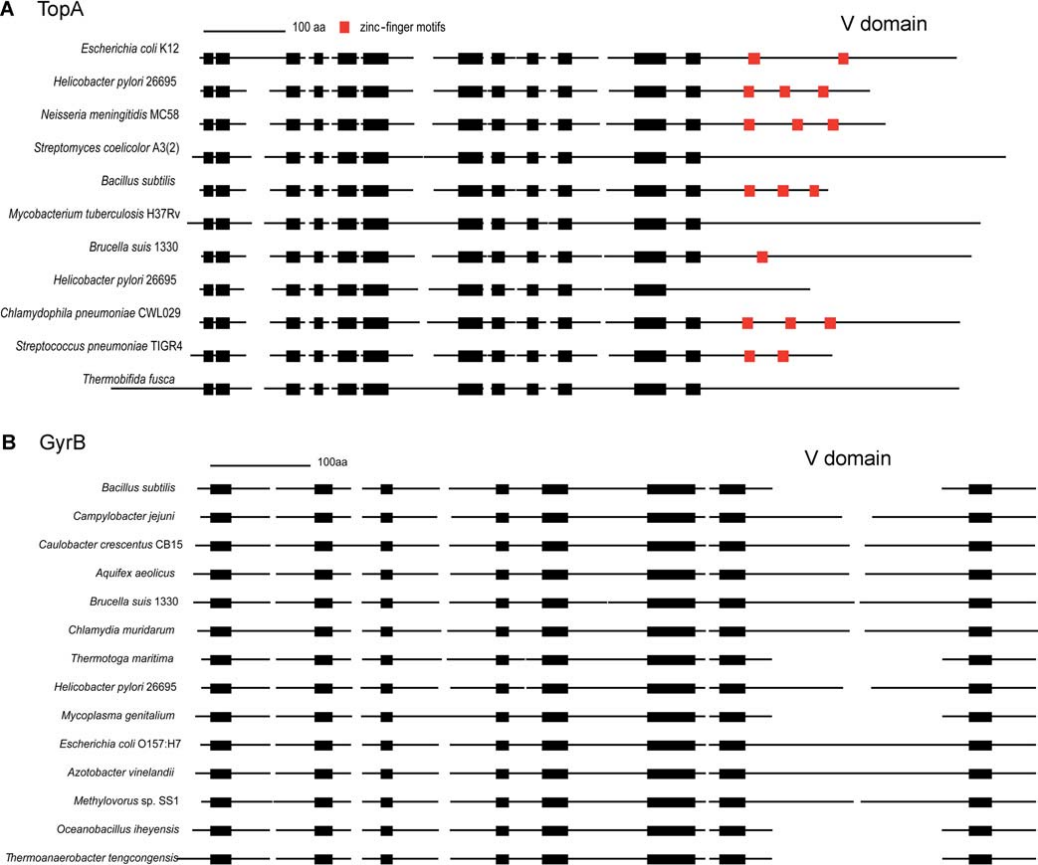
Schematic Alignment of TopA and GyrB (A) TopA. (B) GyrB. Each line represents a sequence. Black boxes indicate the conserved blocks from the BLOCKS database and are aligned correspondingly. Red boxes in (A) are the zinc-finger motifs reported by Pfam. Notice that the number of occurrences of this motif varies and that there are several sequences without this motif in the C-terminal. The lines between the boxes are the variable sequences that cannot be aligned. Variable domains are labeled in the figure.

DNA/RNA processing enzymes form another large family of SVGs. Characteristic examples are the restriction and modification enzymes, where the DNA methylases have a variable region designed for DNA sequence recognition (Cheng 1995) and the restriction enzymes are almost completely variable. Here we discuss two other genes: DNA gyrase B (*gyrB*) and DNA topoisomerase A (*topA*), whose competing actions control the degree of DNA supercoiling (Tse-Dinh et al. 1997). Schematic alignments anchored by the conserved motifs from the BLOCKS database (Henikoff et al. 1999) for both enzymes are shown in Figure 4. The variable region in GyrB is an additional approximately 160 amino acids long segment that is only present in the gram-negative eubacteria (Figure 4B). Experiments probing the role of this region in E. coli GyrB have demonstrated its involvement in DNA binding, although the detailed function is unknown (Chatterji et al. 2000). We suspect that variability in this inserted domain may determine the specificity of the interaction between GyrB and DNA or suggest interaction with other molecules. It is intriguing to see that other gyrases lacking this region are also functional.

For TopA, the N-terminal region of approximately 600 amino acids shows extensive sequence similarity while the C-terminal region (over 100 amino acids) is variable both in sequence content and in length (Figure 4A). The conserved N-terminal region of TopA has the catalytic function of relaxing negatively supercoiled DNA (Feinberg et al. 1999). The variable C-terminus of TopA sometimes contains multiple copies of zinc-binding motifs, although there are a few exceptions, e.g., TopA in Mycobacterium tuberculosis (Figure 4A). Interestingly, there are two copies of TopA in H. pylori 26695; one has three zinc-binding motifs in C-terminal region and the other does not. The zinc-binding motifs in E. coli TopA are shown to be involved in the interaction with the β′ subunit of RNA polymerase (Cheng et al. 2003) and in DNA binding (Ahumada and Tse-Dinh 1998). Since RNA polymerase β′ subunit is a fully conserved gene, the overall sequence variation in the C-terminal region of TopA seems more likely to relate to DNA binding. TopA plays an important role in adaptation to environmental challenges, such as heat shock conditions (Tse-Dinh et al. 1997). Deletion experiments show that in E. coli the C-terminal region is important for the in vivo function of TopA during the osmotic stress response (Cheng et al. 2003). All together, these facts suggest a versatile role that the C-terminal region of TopA might play in those processes.

Variable regions are sometimes found in DNA processing enzymes with essential and conserved functions. One example is DNA polymerase I, which has a variable region between the conserved C-terminal 5′–3′ polymerase domain and the N-terminal 5′–3′ exonuclease domain. In some polymerases, this region encodes a 3′–5′ exonuclease activity for proofreading replication errors, and conserved motifs can be observed (Derbyshire et al. 1995). However, other polymerases in the same family that lack such proofreading activity show much sequence divergence in this region (Derbyshire et al. 1995). The exact reason why sequence variability is observed in these polymerases is unknown.

Another interesting family is the aminoacyl-tRNA synthetases (AARS) (Ibba and Söll 2000). This family of genes is well known for its precision in substrate selection. The molecules known to interact with AARS include tRNA, amino acids, and ATP. Since the same amino acids and ATP molecules are found in all organisms, variability inside the AARS sequences must relate to the recognition and interaction with the tRNAs. Correspondingly, each AARS usually contains a conserved domain for catalysis and acceptor helix interaction and a nonconserved domain that interacts with the variable distal parts of its substrate tRNA (Schimmel et al. 1993). For instance, in bacterial-type prolyl-tRNA synthetase (ProRS), the N-terminal catalytic domain (approximately 200 amino acids) and the C-terminal anticodon-binding domain (approximately 150 amino acids) are highly conserved, while a less conserved region of about 180 amino acids is inserted between them (Figure S2). This variable domain shows similarity to the YbaK domain, which is thought to be involved in oligonucleotide binding (Zhang et al. 2000). Sporadic conserved residues in this region of E. coli ProRS are known to be involved in the posttransfer editing for mischarged Ala-tRNA^Pro^ (Wong et al. 2002). ProRS is also known to possess an inherent ability to mischarge cysteine (Ahel et al. 2002). Partial deletion of this variable region of E. coli ProRS results in a lower rate of proline acylation to cysteine acylation (Ahel et al. 2002), suggesting a possible role of substrate discrimination in this region. Thus, the variability in this inserted domain of ProRS appears to contribute to substrate recognition and the editing function of the enzyme. Intriguingly, ProRS in *Methanococcus jannaschii,* which does not have this inserted region, also possesses editing abilities (Beuning and Musier-Forsyth 2001). As a result, there is a possibility that this region may have another unknown function, e.g., interaction with other undetected molecules.

#### Carbohydrate active enzymes

Variable regions exist in carbohydrate metabolizing enzymes, such as glycosyltransferases (GTs) and glycoside hydrolases (GHs), which respectively catalyze the biosynthesis of diverse glycoconjugates and their selective cleavage (Bourne and Henrissat 2001). Many pathogens express outer-membrane glycosylated oligosaccharides, which closely interact with the host environment (Saxon and Bertozzi 2001). For example, they even mimic host cell surface glycoconjugates to evade immune recognition (Persson et al. 2001). Both GTs and GHs have been classified into subfamilies based on sequence similarity (Bourne and Henrissat 2001). Structural studies on bacterial GTs from different subfamilies always reveal two-domain molecules, such as LgtC (Persson et al. 2001), GtfB (Mulichak et al. 2001), MurG (Hu et al. 2003), and SpsA (Charnock and Davies 1999), with one domain responsible for donor molecule (usually nucleotide-diphospho-sugar) binding and the other domain involved in acceptor sugar molecule binding. These genes exhibit great variability in the acceptor-binding domains and conservation in the donor-binding domains (see Figure S3 for the example of GtfB), which agrees with the relatively limited types of donor species (usually UDP/TDP-sugar) and their conserved binding modes, but a diversity of acceptor molecules (LgtC: lactose; GtfB: vancomycin aglycone; MurG: *N*-acetyl muramyl pentapeptide; SpsA: unknown). Owing to the lack of homology in the acceptor binding domains, the substrate specificities encoded by these regions for most GTs are still unknown.

#### Transcriptional regulators

Prokaryotic transcriptional regulators form another large group of SVGs. Transcription regulators are usually two-domain proteins with one binding to DNA and one binding to ligand. The DNA-binding domains, which usually interact with DNA via helix–turn–helix, zinc-finger, or other modes, are more conserved than ligand-binding domains. Based on the characteristic conserved DNA-binding domains, transcriptional regulators can be classified into many different families (Nguyen and Saier 1995; Rigali et al. 2002). Even within each family, the ligand-binding domains are variable. For instance, the C-terminal regions involved in effector molecule binding and oligomerization (E-b/O) inside the GntR transcriptional regulator family are highly variable both in sequence content and in size (Rigali et al. 2002). The variability in the effector molecule-binding domains enables the transcriptional regulators to sense the presence of diverse ligands and signal the regulation of the downstream genes or operons accordingly. As in most previous cases, these variable regions remain functionally uncharacterized.

#### Hypothetical genes

In addition to genes with functional annotations, our method identifies a number of SVGs with unknown or hypothetical annotations in each genome (H. pylori: 17 genes; N. meningitidis: 32 genes; V. cholerae: 69 genes, etc.; see http://geneva.bu.edu for the full list). In contrast to lineage-specific hypothetical genes, these hypothetical genes contain conserved domains, which suggest their functional importance. Although most of the conserved domains in these hypothetical genes have currently unknown function, there are a few exceptions. Among them are the prokaryotic mechanosensitive channel proteins, which respond to external osmotic pressure (Pivetti et al. 2003). Examples include the 343 amino acid long E. coli B1330 and 371 amino acid long Bacillus subtilis YhdY, both of which are currently annotated as “hypothetical.” However, they both have the characteristic domain of mechanosensitive proteins (Pfam domain: MS_channel). The central regions (approximately 150 amino acids) of these genes are conserved while both the N-terminal region (approximately 100 amino acids) and the C-terminal region (approximately 100 amino acids) are variable (see alignment in Figure S4). The conserved central region encodes three transmembrane segments, and the molecules are predicted to have their N-terminus outside and C-terminus inside the cell (Miller et al. 2003). Although the C-terminus is variable, the deletion experiments show that it is indispensable for stability and activity of this protein (Miller et al. 2003). It is tempting to hypothesize that the interacting partners for both N- and C-termini might vary in different organisms.

### Functional Classification of SVGs

**Figure 5:**
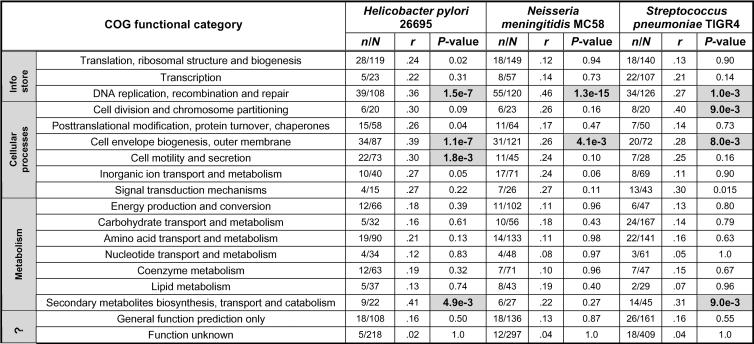
Functional Classification of SVGs in Three Microorganisms *M* is the total number of genes in a COG broad functional category, and *m* is the number of SVGs within that category. *r* ( = *m/M*) is the proportion of SVGs in that category. The *p*-value is calculated using a hypergeometric distribution: let *N* = number of genes in the genome; *n* = number of SVGs identified; *M* = number of genes belonging to a particular category; *m* = number of SVGs belonging to a particular category: 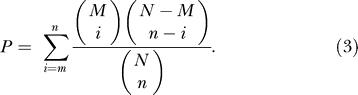 The set of lineage-specific genes has been excluded in each genome to avoid the possible skew it brings to the estimation of significance. The significance level is set at 0.01. Cells with *p*-value less than 0.01 are shaded.

We are interested in probing the functional distribution of SVGs within a single genome. Are certain functional categories overrepresented? In Figure 5, we show a functional classification of SVGs in three microorganisms using 18 broad functional categories of the clusters of orthologous group (COG) database (Tatusov et al. 1997). We calculated the percenta*g*e (*r* in Figure 5) of SVGs within each functional class and the *p*-value of overrepresentation (Figure 5). Several functional categories are overrepresented (*p*-value < 0.01; see Figure 5 for details): (i) cell envelope biogenesis, outer membrane; (ii) DNA replication, recombination and repair; (iii) secondary metabolite biosynthesis, transport and catabolism; (iv) cell motility and secretion; (v) cell division and chromosome partitioning. Among them, only categories (i) and (ii) are overrepresented in all three genomes. Most functional categories involved in the basic metabolic processes are not significantly overrepresented or even underrepresented. The number of overrepresented categories and the order of significance differ from one genome to another, reflecting differences in genome content and presumably the relative importance of the different specific adaptations.

**Figure 6:**
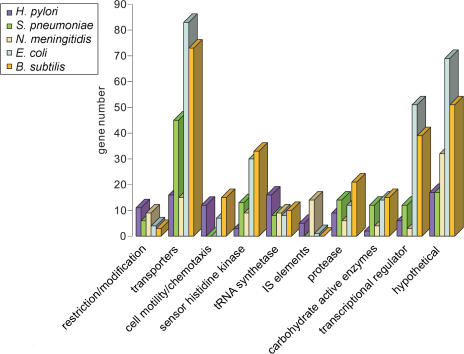
Abundance of SVGs in Different Functional Categories in Five Microorganisms The approximate total gene number for each organism is as follows: H. pylori, 1,566 genes; S. pneumoniae, 2,094 genes; N. meningitidis, 2,065 genes; E. coli, 4,289 genes; B. subtilis, 4,100 genes.

In Figure 6 we show the relative abundance of a set of SVG families in several microorganisms based on shared keywords in the annotations. The relative enrichments in several gene families for some microbes seem to correlate with the peculiarities of niche adaptation. In particular, H. pylori has more SVGs involved in cell motility and chemotaxis than two other genomes with a similar genome size *(N. meningitidis, Streptococcus pneumoniae). H. pylori* is one of the few microbes that can colonize the highly acidic gastric environment (Tomb et al. 1997). The motility of H. pylori is crucial for its infectious capability and there is evidence that poorly motile strains are less able to colonize or survive in the host (O'Toole et al. 2000). S. pneumoniae has more carbohydrate-metabolizing enzymes, especially glycosyltransferases (GTs), which appear to be segmentally variable. The unique pattern of cell surface glycosylation in S. pneumoniae has been under extensive investigation and plays an important role in pathogenesis (Tettelin et al. 2001). The GTs are responsible for making *O*-linked glycosylations on surface proteins, which coat the surface of the bacterium and interact with the host (Tettelin et al. 2001).

### Gene Duplication and SVGs

Duplication followed by diversification is an efficient way of generating functional innovations (Prince and Pickett 2002). Regional sequence divergence has been observed between duplicated gene copies (Gu 1999; Dermitzakis and Clark 2001; Marin et al. 2001). We thus asked the following questions: (1) What is the distribution of paralogous genes in the set of SVGs in a single genome? (2) Is there a significant association between gene duplication and SVGs?

**Figure 7:**
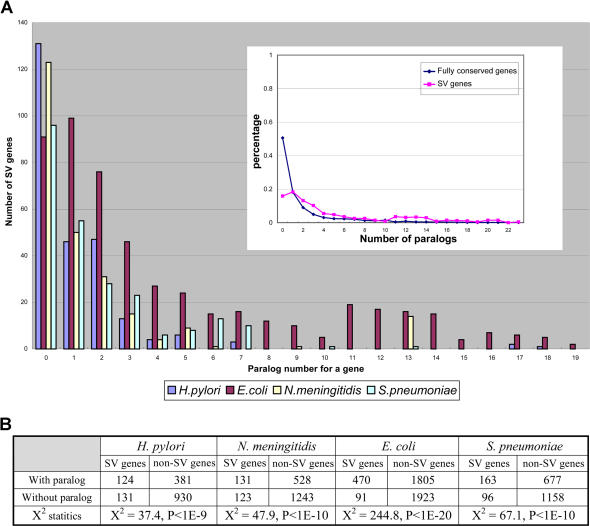
Paralogous Genes in SVGs (A) Paralog families in SVGs for four microorganisms. The x-axis shows the number of paralogs for each SVG. The y-axis shows the number of SVGs. The inset figure shows the percentage of genes with different numbers of paralogs for SVGs and fully conserved genes in E. coli genome. The x-axis is the number of paralogs, and the y-axis is the percentage. (B) Contingency tables to examine the dependence between SVG and paralogous gene. χ^2^ statistics are computed using standard formula.

In Figure 7A, we show the distribution of paralogous genes among SVGs in several genomes. We consider paralogous genes to be similar genes in the same genome with a BLAST *E*-value less than 1*E*-5. As shown in Figure 7A, in H. pylori, *N. meningitidis,* and S. pneumoniae, the largest group of SVGs is the one with no paralogs. However, in E. coli, the largest group is the one with a single paralog. E. coli obviously has more paralogous genes in the SVG set, probably owing to a larger genome size by duplication. In Figure 7A (inset), we show the percentage of genes with different numbers of paralogs in each class for both segmentally variable and fully conserved genes in E. coli. Interestingly, over half of the fully conserved genes in E. coli do not have paralogs. There is a significant difference between the two distributions (χ^2^ test, *p*-value < 1*E*-5). In Figure 7B, we list the number of genes in a contingency table and test the significance using a χ^2^ test. For all genomes examined, there is a strong association between gene duplication and SVGs, suggesting an SVG is more likely to have originated from a duplicated gene.

Here we give an interesting example where one paralogous copy of a gene is segmentally variable and the other copy is fully conserved. In H. pylori strain 26695, gene products of *HP1299* (253 amino acids) and *HP1037* (357 amino acids) both have a conserved domain (approximately 250 amino acids; Pfam: Peptidase_M24) that is characteristic of the methionyl aminopeptidase (*map*) family (metalloprotease family M24) (Rawlings and Barrett 1995). *HP1299* is fully conserved in a number of microbes and is homologous to the *E. coli map* gene (Figure S5), while the product of *HP1037* has an extra N-terminal region (approximately 100 amino acids) that is variable among its similar genes (Figure S6). Additionally, *HP1037* is annotated as a conserved hypothetical gene. The five residues found in the *E. coli map* that are involved in cobalt (Co^2+^) binding (Asp-97, Asp-108, His-177, Glu-204, Glu-235; Rawlings and Barrett 1995), are conserved in both genes by examining the multiple alignment. These findings suggest that *HP1037* might also encode a *map* activity and that its variable N-terminal region might be involved in additional functional roles, e.g., interactions with other molecules. In Saccharomyces cerevisiae, there are two *map* genes and both have an extra N-terminal region compared to the *E. coli map* gene. One copy of the yeast *map* gene contains zinc-finger motifs in the N-terminal region that are indispensable for in vivo function (Li and Chang 1995). A functional role involving interaction with the ribosome has also been suggested for this N-terminal domain (Vetro and Chang 2002). In most prokaryotes, it has been assumed that there is only one copy of the *map* gene. The SVG family exemplified by *HP1037* may represent another family of *map* genes in prokaryotes.

## Discussion

A major fraction of bioinformatics research on sequence analysis has focused on the conserved regions in proteins, trying to hypothesize the role of the protein by identifying sequence motifs that have been shown experimentally to correlate with a specific function. Some work has gone into cataloging the groups of lineage-specific proteins that show no similarity to other proteins in GenBank (Galperin and Koonin 1999), but there the route to assigning function usually needs experimental approaches requiring biochemistry or genetics or more rarely by determining the crystal structure of the gene product (Zhang et al. 2000). Unfortunately, current bioinformatics methods are only occasionally helpful in suggesting where to begin such studies.

In this paper we have initiated an effort to identify SVGs, which contain both well-conserved regions and highly variable regions. By looking carefully at a few specific examples where functional information is available from experimental data, we find that the variable region often seems to play a key role in mediating interactions with other molecules, both large and small. Sometimes the variable portions are involved in biological processes with a component of interaction between the cell and agents from the external environment. For instance, the DNA methyltransferases are part of a defense system that recognizes and clears invading foreign DNA; membrane-bound sensory HKs and mechanosensitive ion channels, etc., monitor changes of living conditions. Sometimes the variable portions are involved in intracellular processes that appear to have lineage-specific features. Thus, the variable regions inside DNA GyrB and several types of AARSs probably determine the specificity of substrate recognition. The detailed factors that introduce the molecular variability may go well beyond our explanations here and likely vary from case to case. Some variable regions may have diverged a long time ago and are now kept constant, while others may keep changing. In all of these cases, SVGs are exceptionally worthy targets of further experimental investigation, and such investigations can be greatly aided by the presence of the conserved regions that may suggest a preliminary function to be tested.

Why might certain genes contain these variable regions? Could they be simply relics left over during evolution and now serve no purpose? Are they just “pseudo-segments” with no function? There are several lines of evidence that support the hypothesis that when variable regions have been retained, they indeed serve a function. First, several studies have shown that deletions are, on average, more frequent than insertions (Halliday and Glickman 1991). As a result, if a region is evolving under weak functional constraints, it tends to get smaller over time (Lipman et al. 2002). Second, in a special case, one can imagine that when a variable region occurs at the C-terminus of a protein and is not being selected, it is likely to suffer random mutations including nonsense mutations or insertions/deletions that cause a shift in reading frame. Thus, we searched GenBank release 136.0 for examples of genes that matched the conserved region of an SVG, but in which the C-terminus was missing or much shorter. The DNA sequences downstream of such hits were examined for similarity to the variable region in the query gene. Of the 83 SVGs with a C-terminal variable region in H. pylori, none of them had hits with a disrupting stop codon in the variable region; 20 of them have hits with genes showing insertions/deletions that cause frame shifts in the variable region. However, the real number is likely to be much fewer, since, based on previous work, many of them may be the results of sequencing errors (Posfai and Roberts 1992).

In other cases, we find that some proteins have lost the variable segment in a subset of genomes. For instance, in ProRSs, the variable segment is absent in archaea; in GyrB, the variable segment is absent in the Gram-positive bacteria. Clearly in those cases the organisms can get by without the variable domain, although they may have a compensating function in a different gene. But this again does not imply that the variable region has no function in those genes that have retained it.

SVGs are distinct from sequences with shuffled domains (Doolittle 1995) in that the variable region is bounded by the same sets of conserved portions, while domain shuffling usually manifests itself in a different sequential order of conserved domains. We also hypothesize that the variable regions in SVGs are not the result of multiple domain fusion events, each resulting in an insertion of a different sequence into the protein. This hypothesis is supported by the fact that the fused domains are often conserved across multiple organisms (Marcotte et al. 1999). Additionally, our procedure requires that the variable regions are of similar length within a family of proteins, which are also restricted to conserved length distributions. This filter suggests a mutational mechanism that originated from an ancient protein. Indeed, it is possible that originally the variable region was a result of a single or possibly relatively few ancient fusion events, but this paper does not focus on the evolutionary origin of SVGs.

Another prediction from our observations is that the variable regions are excellent candidates to bind substrates or partner macromolecules. They may be extremely helpful in discovering the networks of protein–protein or protein–nucleic acid interactions within a cell. Bioinformatics may even be able to help in this endeavor by finding genes that seem to have coevolving variable regions as a result of such interactions. Experimental data from techniques such as the yeast two-hybrid system or microarrays may provide evidence for interactions that can involve two variable regions.

Much additional bioinformatics work will be needed to explore fully the potential of this method in hypothesizing function. For instance, the size limits we have arbitrarily imposed on the variable region should be tested systematically. In our relatively simple formulation presented here, the length of the variable region and the number of proteins in the same family that do not have an alignment to the variable region are the primary factors in determining its statistical significance. Methods using other sequence analysis tools, such as multiple alignment and sequence profiles, may provide alternative ways to identify segmental pattern of variability. A fundamental problem is to differentiate random evolutionary drift from positive selection correlated to functional requirements. Although one might expect that the N- and C-termini may be more variable than the regions in the middle, our data suggest that variable regions in SVGs are not preferentially located in either end (data not shown). We have also examined the amino acid composition, codon usage, and GC content in the variable regions and the conserved regions of the same SVG. While there is no significant deviation of amino acid composition and GC content between the two regions in general, codon usage appears to be biased in the variable regions (data not shown).

SVGs usually account for 10%–20% of the total genes in a microbial genome. Currently, we think of the class of lineage-specific genes as being the key factor that distinguishes one strain or species from another. The class of SVGs that we have defined in this paper must now be added to this collection of lineage-specific genes by virtue of the unique segments that constitute their variable regions. They also appear to provide functional elements that help to differentiate among strains and species. This point is well illustrated by considering the restriction-modification systems. Here, the DNA methyltransferases, which have a variable region responsible for DNA recognition, are members of the SVG class. With the help of their companion restriction endonucleases, which typically appear as lineage-specific genes, they serve to keep foreign, unmodified DNA sequences from entering the genome. In this case, the synergy of function provided by members of the two classes highlights the key role that both sets of genes must play in defining the individuality of a strain or species.

Our analysis to date is limited to prokaryotes and archaea where SVGs are transcribed and translated as contiguous genomic segments. In eukaryotes, alternative RNA splicing introduces substantial additional complexity into the interpretation of gene structure and protein product, thereby rendering impossible the simple analysis we have applied here. It is tempting to consider alternative splicing as a highly evolved control mechanism to introduce the variability we find in the SVGs and thereby achieve the functional diversity necessary for cell survival under different conditions. In eukaryotes, alternatively spliced exons can be introduced in response to the functional demands of different cell types by merely juggling protein coding regions in the genome, thereby creating an SVG structure. If this view is correct, then it reinforces and highlights the importance of these SVGs to the workings of the cell.

In this paper we have provided an initial glimpse of SVGs, which appear to provide an important genetic layer in the adaptation of cells to novel environments and hazardous pathogens. We have focused attention on the biological significance of these genes, especially those that have highly diverged segments. We are currently trying to develop a more refined classification of these genes so as to explore the functional significance of the variability. We would like to know whether extreme variability is required for diverse function or whether more modest variation is sufficient. Such questions require that we can first distinguish positive selection acting on these variable regions from neutral evolution leading to gene decay and eventual loss. Since the variable regions we report are often not amenable to current tools available for alignment, we are exploring new methods that will help us to assess whether positive selection is driving the evolution of these genes.

In summary, we have identified an extremely useful way of classifying genes that leads to the identification of those with a high priority for both experimental and computational research.

## Materials and Methods

### 

Our method for detecting SVGs includes several steps: (1) identification of similar genes followed by query-anchored multiple alignment using nongapped BLAST (Altschul et al. 1990); (2) taxonomy clustering of similar genes to avoid bias; (3) detection of segmental variability.

#### Identification of similar genes

Given a gene, we start by searching for all its similar genes in the nonredundant database (GenBank release 136.0, 15 June 2003) using nongapped BLAST (Altschul et al. 1990). We use the nongapped BLAST because the gapless high scoring pairs (HSPs) reported are rather conservative. The gapped BLAST, however, tends to extend HSPs over variable regions, which has been observed in several examples (e.g., DNA-recognition domain in cytosine-specific methyltransferase; data not shown). Two criteria are used to define close similarity. First, the *E*-value is less than 1*E*-10. Here we use a strict *E*-value threshold to avoid possible functional divergence among the homologs. Accordingly, we use the BLOSUM80 scoring matrix in the BLASTP search, although the result does not change dramatically if BLOSUM62 is used. Second, the overall length of the hit sequence does not differ significantly from the query sequence. We define the gap content (GapC) between two sequences:



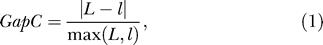



where *L,l* are the lengths of the protein sequences of two genes. It is a measure of the smallest percentage of gaps needed to be introduced into the pairwise alignment. Sequences with a high GapC value indicate significantly different domain structures, possibly owing to domain insertions or losses, and thus are excluded from the set of similar genes. In our current implementation, we require that GapC must be less than 0.2.

#### Taxonomy clustering of the similar genes

Similar genes reported by BLASTP are not evenly distributed among different species. In many cases, highly similar genes from different strains of the same species or highly similar paralogous genes from a particular strain tend to introduce bias into the dataset. We adopted a simple taxonomy clustering by using the NCBI Taxonomy Database (Wheeler et al. 2003) to reduce this bias.

We collapse all the similar genes from the same species into a single group. Then we choose the gene with the best similarity score to the query sequence as the representative of that species for later calculations. The definition of species follows the hierarchical taxonomy used in the NCBI Taxonomy database (superkingdom → phylum → class → subclass → order → family → genus → species → no rank [strain]). By doing taxonomy clustering, we are able to collect a less biased sample of similar genes from different species.

#### Detection of segmental variability

Query-anchored multiple alignment after taxonomy clustering is performed by aligning the HSPs reported by nongapped BLAST (see Figure S2 and http://geneva.bu.edu). Two unaligned regions in two sequences are considered as the variable regions if they are bounded by similar HSPs at both ends (or one end, if the unaligned region is at the terminus of the gene). To avoid the possibility of a large segment containing insertions or deletions, we again require that GapC be less than 0.2 between these two unaligned regions.

For each amino acid position in the query gene, we can count the number of times *(m)* it is inside an HSP region and the number of times *(n)* it is inside a variable region. A high ratio of *n* over *m* + *n* suggests that this position is inside the variable region most of the time. We estimate the statistical significance (*p*-value) of the variability for each position by a binomial distribution:



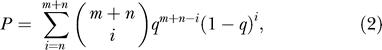



where *q* is the probability of an amino acid position being inside a HSP region. We estimate *q* by averaging the proportion of HSP in each hit sequence among all hits. If the *p*-value calculated using the above formula is less than the significance level, which we set at 0.05, we then consider this position as a variable position; otherwise, it is a conserved position. A consecutive run of variable positions forms a variable region. The next question is how long the variable region should be to be considered meaningful, as opposed to functionally unimportant regions such as linker regions, which are usually short. From our experience, there is no clear decision boundary between the length of the region and its functional importance. Any choice of cutoffs would have to balance between false positives and false negatives. However, previous studies on the length distribution of protein domains has shown that the most likely length of a protein domain is around 70 amino acids, and regions shorter than this are less likely to form a functional domain (Wheelan et al. 2000). Based on this, we chose 70 amino acids as the length threshold for a variable region to be considered functionally important. In Figure S7, we show the length distribution of the variable regions in all genes of *H. pylori.*

A direct way of visualizing the variability of a protein sequence is by calculating the ratio of *n* over (*m* + *n*) for each position and plotting it. We call such plots variability profiles. Sample variability profiles are shown in Figure 1. In Figure 1A, two obvious peaks are present: one from position 20 to 70, the other from position 160 to 300. The latter (approximately 140 amino acids) forms a separate DNA recognition domain, while the former (approximately 50 amino acids) has no known function. In Figure 1 we also show conserved subsequences from the Pfam (Bateman et al. 2002), BLOCKS (Henikoff et al. 1999), and PRINTS (Attwood et al. 2003) databases. The BLOCKS and PRINTS databases are relatively conservative in defining motifs. However, the Pfam domain seems to include the variable region within the conserved region, as shown in Figure 1A.

## Supporting Information

### Data Deposit

We provide a static collection of segmentally variable genes at our Web site, http://geneva.bu.edu. SVGs for several representative genomes are listed there. For SVG lists in other genomes, please request more information from Y. Zheng at E-mail: zhengyu@bu.edu. All the case examples mentioned throughout the paper and Supporting Information have been compiled into one Web page, http://geneva.bu.edu/paper03.html, with hyperlinks. Readers can follow each hyperlink to access additional information from Pfam, BLOCKS, PRINTS, COG, and nongapped BLAST for each gene.

Figure S1 Multiple Alignment of BtuB and Homologs Conservation score is plotted under the alignment (ClustalX). The conserved portions are as follows: N-terminal domain, extreme C-terminal domain, and a segment between N-terminal and C-terminal domain. The variable domain (between N-terminal and C-terminal) overlaps with the transmembrane 22-strand β-barrel regions.
(2.69 MB EPS).

Figure S2 Query-Anchored Alignment of ProRS The query protein is H. pylori ProRS. The blue segments are HSPs reported by nongapped BLAST. The yellow segments are the variable region. The gray region is the gap-rich region (GapC > 0.2, deletion in this alignment). See http://geneva.bu.edu/paper03.html for a high-resolution Web figure.
(4.71 MB EPS).

Figure S3 Multiple Alignment of GtfB and Its Homologs (3.12 MB EPS).

Figure S4 Multiple Alignment of B. subtilis Gene *yhdY* and Its Homologs YhdY is currently annotated as a hypothetical protein and contains a conserved domain for mechanosensitive proteins (the middle region of the alignment) and two variable domains (N- and C-termini).
(2.86 MB EPS).

Figure S5 Multiple Alignment for H. pylori Gene *HP1299*
It is the methionine aminopeptidase (type Ia *map*). This is an example of a fully conserved gene.
(1.87 MB EPS).

Figure S6 Multiple Alignment for H. pylori Gene *HP1037*
It is currently annotated as “conserved hypothetical protein.” The N-terminal region is variable. The conserved C-terminal domain is characteristic of methionine aminopeptidase.
(2.22 MB EPS).

Figure S7 Length Distribution of Variable Regions in the Genome of H. pylori
Shown as a histogram. Only variable regions inside fully conserved genes and SVGs are included. Pink line shows the domain size distribution in 3D-structure database (data from Wheelan et al. 2000).
(643 KB EPS).

Table S1 Classification of Genes into Three Broad Categories (62 KB DOC).

### Accession Numbers

The GenBank (http://www.ncbi.nlm.nih.gov/GenBank/) accession numbers for the genes discussed in Figure 3 are as follows: *atpA* (2314285), *atpD* (2314283), *atpG* (2314284), *dnaX* (2313841), *flgK* (2314271), *ftsK* (2314237), *gyrB* (2313611), *hetA* (2314367), *HP1450* (2314626), *infB* (2314195), *M.hpyAVIB* (2313124; REBASE [http://rebase.neb.com] ID M2.hpyAVI), *mutS* (2313742), *NQO3* (2314431), *NQO8* (2314432), *polA* (2314647), *rps4* (2314460), *spaB* (2313717), *spoT* (2313901), *tlpA* (2313179), and *tlpC* (2313162).

The GenBank accession numbers for the genes discussed in Figure 3 are as follows: Agrobacterium tumefaciens (15890351), B. subtilis (16079962), Enterococcus faecalis (8100675), E. coli K12 (16128553), L. innocua (16801788), Mycobacterium leprae (15826988), M. tuberculosis CDC1551 (15840173), Nitrosomonas europaea (22955201), Nostoc sp. PCC 7120 (17228666), P. syringae pv. syringae B728a (23470301), Ralstonia metallidurans (22980570), R. solanacearum (17548875), Synechococcus sp. PCC 7942 (21954778), and Thermotoga maritime (15644402); in case studies, B. subtilis yhdY (2633299), E. coli b1330 (1787591), H. pylori cytosine-specific DNA methyltransferase (2313124), H. pylori HP1299 (2314463), H. pylori HP1037 (2314181), H. pylori prolyl-tRNA synthetase (2313329), and H. pylori VacB (2314413).

## Abbreviations

AARS: aminoacyl-tRNA synthetase
ABC transporter: ATP-binding cassette transporter
COG: clusters of orthologous group
2D: two dimensional
3D: three dimensional
E-b/O: effector molecule binding and oligomerization
GapC: gap content
GH: glycoside hydrolase
GT: glycosyltransferase
GyrB: DNA gyrase B
HK: histidine kinase
HSP: high scoring pair
*map*: methionyl aminopeptidase
MCP: methyl-accepting chemotactic protein
ProRS: prolyl-tRNA synthetase
rRNA: ribosomal RNA
SVG: segmentally variable gene
TopA: DNA topoisomerase A
