# Supplementary material for: Segmentally Variable Genes:A New Perspective on Adaptation
Source: PLoS Biol. 2004 Apr 13;2(4):e81. doi: 10.1371/journal.pbio.0020081 (PMC387263; doi:10.1371/journal.pbio.0020081)
Supplement: Table S1 — (62 KB DOC). [file pbio.0020081.st001.doc]

**Table S.1 Classification of genes into three broad categories**

| **Microorganism** | | **Partially variable genes** | | | | **Fully-conserved genes** | **Species-specific genes** |
| --- | --- | --- | --- | --- | --- | --- | --- |
|  | | **Total** | variable domain = 1 | variable domain = 2 | variable domain > 2 |  |  |
| Bacteria | *Helicobacter pylori 26695* | 255(16%) | 210 | 37 | 8 | 958(62%) | 353(22%) |
|  | *Neisseria meningitidis MC58* | 254(12%) | 226 | 22 | 6 | 1373(67%) | 440(21%) |
|  | *Streptococcus pneumoniae TIGR4* | 259(12%) | 220 | 34 | 5 | 1485(71%) | 357(17%) |
|  | *Escherichia coli* | 561(13%) | 488 | 66 | 7 | 3581(83%) | 147(3%) |
|  | *Bacillus subtilis* | 535(13%) | 476 | 49 | 10 | 3214(78%) | 351(9%) |
|  | *Streptomyces coelicolor* | 1111(14%) | 940 | 146 | 25 | 5821(74%) | 965(12%) |
|  | *Mycoplasma genitalium* | 99(20%) | 85 | 11 | 3 | 380(79.8%) | 1(0.2%) |
|  | *Buchnera sp* | 96(16%) | 81 | 15 | 0 | 481(83.4%) | 4(0.6%) |
|  | *Haemophilus influenzae* | 197(12%) | 169 | 25 | 3 | 1447(84%) | 65(4%) |
|  | *Bacillus anthracis Ames* | 579(11%) | 518 | 52 | 9 | 3895(73%) | 837(16%) |
|  | *Staphylococcus aureus Mu50* | 377(14%) | 340 | 30 | 7 | 1847(71%) | 400(15%) |
|  | *Brucella suis 1330* | 406(12%) | 365 | 38 | 3 | 2312(71%) | 546(17%) |
|  | *Vibrio cholerae* | 545(14%) | 472 | 63 | 10 | 2775(73%) | 508(13%) |
|  | *Salmonella typhimurium* | 456(15%) | 395 | 56 | 5 | 3728(85%) | 211(5%) |
|  | *Pseudomonas aeruginosa* | 847(15%) | 744 | 93 | 10 | 4374(79%) | 344(6%) |
|  | *Deinococcus radiodurans* | 423(14%) | 374 | 47 | 2 | 1878(60%) | 801(26%) |
|  | *Tropheryma whipplei Twist* | 130(16%) | 111 | 17 | 2 | 549(68%) | 129(16%) |
|  | *Lactobacillus plantarum* | 433(14%) | 372 | 57 | 4 | 2150(72%) | 426(14%) |
|  | *Coxiella burnetii* | 165(8%) | 149 | 13 | 3 | 1181(59%) | 663(33%) |
|  | *Nitrosomonas europaea* | 320(13%) | 276 | 37 | 7 | 1885(77%) | 256(10%) |
|  | *Chlamydophila caviae* | 145(14%) | 121 | 23 | 1 | 786(79%) | 74(7%) |
|  | *Wigglesworthia brevipalpis* | 109(18%) | 98 | 10 | 1 | 496(81%) | 6(1%) |
|  | *Bifidobacterium longum* | 336(19%) | 283 | 43 | 10 | 1129(66%) | 264(15%) |
|  | *Bacteroides thetaiotaomicron VPI-5482* | 571(13%) | 436 | 110 | 25 | 2817(58%) | 1390(29%0 |
|  | *Vibrio parahaemolyticus* | 618(13%) | 536 | 71 | 11 | 3416(70%) | 798(17%) |
|  | *Leptospira interrogans* | 498(11%) | 413 | 71 | 14 | 1888(39%) | 2341(50%) |
|  | *Agrobacterium tumefaciens C58* | 832(16%) | 741 | 78 | 13 | 3964(74%) | 505(10%) |
|  | *Shigella flexneri 2a* | 402(10%) | 358 | 42 | 2 | 3713(88%) | 65(2%) |
|  | *Mycobacterium leprae* | 233(15%) | 185 | 38 | 10 | 1257(78%) | 115(7%) |
|  | *Oceanobacillus iheyensis* | 478(14%) | 428 | 420 | 8 | 2663(76%) | 355(10%) |
|  | *Ureaplasma urealyticum* | 102(17%) | 84 | 16 | 2 | 342(56%) | 167(27%) |
|  | *Sulfolobus solfataricus* | 333(11%) | 285 | 43 | 5 | 2307(78%) | 337(11%) |
|  | *Shewanella oneidensis* | 506(11%) | 439 | 56 | 11 | 3174(66%) | 1098(23%) |
|  | *Synechocystis PCC6803* | 424(13%) | 351 | 64 | 9 | 2521(80%) | 224(7%) |
|  | *Streptococcus pyogenes* | 225(13%) | 186 | 31 | 8 | 1349(80%) | 122(7%) |
| Archeae | *Pyrococcus horikoshii* | 178(9%) | 152 | 23 | 3 | 1494(72%) | 392(19%) |
|  | *Methanococcus jannaschii* | 178(10%) | 146 | 27 | 5 | 1263(71%) | 329(19%) |
|  | *Methanopyrus kandleri* | 150(9%) | 131 | 16 | 3 | 1052(62%) | 485(29%) |
|  | *Pyrococcus abyssi* | 184(10%) | 159 | 22 | 3 | 1532(87%) | 49(3%) |
|  | *Aeropyrum pernix* | 245(9%) | 198 | 42 | 5 | 1039(39%) | 1410(52%) |
|  | *Archaeoglobus fulgidus* | 242(10%) | 209 | 27 | 6 | 1697(71%) | 468(19%) |
|  | *Methanobacterium thermoautotrophicum* | 180(10%) | 143 | 32 | 5 | 1351(72%) | 338(18%) |
|  | *Aquifex aeolicus* | 234(15%) | 210 | 22 | 2 | 1101(71%) | 218(14%) |
